# ACE gene dosage determines additional autonomic dysfunction and increases renal angiotensin II levels in diabetic mice

**DOI:** 10.6061/clinics/2018/e246

**Published:** 2018-07-10

**Authors:** Oscar Albuquerque de Moraes, Karin Flues, Kátia Bilhar Scapini, Cristiano Mostarda, Fabiana de Sant’Anna Evangelista, Bruno Rodrigues, Daniela Ravizzoni Dartora, Patricia Fiorino, Kátia De Angelis, Maria Cláudia Irigoyen

**Affiliations:** I Instituto do Coracao (InCor), Hospital das Clinicas HCFMUSP, Faculdade de Medicina, Universidade de Sao Paulo, Sao Paulo, SP, BR; II Laboratorio do Movimento Humano, Universidade Sao Judas Tadeu, Sao Paulo, SP, BR; III Universidade Federal do Maranhao (UFMA), Sao Luiz, MA, BR; IV Escola de Artes, Ciencias e Humanidades, Universidade de Sao Paulo (USP), Sao Paulo, SP, BR; V Departamento de Atividade Adaptada, Universidade de Campinas (UNICAMP), Campinas, SP, BR; VI Instituto de Cardiologia do Rio Grande do Sul, Fundacao Universitaria de Cardiologia (IC/FUC), Porto Alegre, RS, BR; VII Laboratorio de Fisiofarmacologia Metabolica Renal e Cardiovascular, Centro de Ciencias Biologicas e da Saude, Universidade Mackenzie, Sao Paulo, SP, BR; VIII Departamento de Fisiologia, Universidade Federal de Sao Paulo (UNIFESP), Sao Paulo, SP, BR

**Keywords:** Renin-angiotensin System, Autonomic Nervous System, Renal Angiotensin System, Mice

## Abstract

**OBJECTIVES:**

The present study aimed to investigate cardiovascular **autonomic** modulation and angiotensin II (Ang II) activity in diabetic mice that were genetically engineered to harbor two or three copies of the angiotensin-converting enzyme gene.

**METHODS:**

Diabetic and non-diabetic mice harboring 2 or 3 copies of the angiotensin-converting enzyme gene were used in the present study. Animals were divided into 4 groups: diabetic groups with two and three copies of the angiotensin-converting enzyme gene (2CD and 3CD) and the respective age-matched non-diabetic groups (2C and 3C). Hemodynamic, cardiovascular, and autonomic parameters as well as renal Ang II expression were evaluated.

**RESULTS:**

Heart rate was lower in diabetic animals than in non-diabetic animals. Autonomic modulation analysis indicated that the 3CD group showed increased sympathetic modulation and decreased vagal modulation of heart rate variability, eliciting increased cardiac sympathovagal balance, compared with all the other groups. Concurrent diabetes and either angiotensin-converting enzyme polymorphism resulted in a significant increase in Ang II expression in the renal cortex.

**CONCLUSION:**

Data indicates that a small increase in angiotensin-converting enzyme activity in diabetic animals leads to greater impairment of autonomic function, as demonstrated by increased sympathetic modulation and reduced cardiac vagal modulation along with increased renal expression of Ang II.

## INTRODUCTION

The clinical relevance of the angiotensin converting enzyme (ACE) polymorphism was first described in 1990, when Rigat et al. 1 found a 2-fold increase in serum ACE levels in patients with the ACE DD genotype compared with ACE II patients.

In concordance with Rigat et al., in recent years, several studies have demonstrated an association of the DD genotype and the specific D allele with clinical cardiovascular outcomes, such as atherosclerosis 2 and increased left ventricular mass in systemic hypertension 3,4. Moreover, observational data demonstrated an elevated frequency of the ACE DD genotype in type 2 diabetes patients 5.

Randomized clinical trials (RCT) have shown the ability of renin-angiotensin system (RAS) inhibitors to prevent new-onset diabetes mellitus (DM) and its vascular complications 6. Meta-analysis data agree with RCT data, showing a reduced risk of developing DM in patients taking ACE inhibitors or angiotensin II (Ang II) receptor blockers (ARB). However, large double-blind RCTs did not confirm the previous hypothesis; one study showed that ramipril was not able to prevent DM development in patients with impaired fasting glucose metabolism 7.

Ang II influences several physiological domains in organ systems, including the autonomic nervous system (ANS). In fact, Ang II may stimulate the sympathetic system and inhibit parasympathetic outflow. On the other hand, when Ang II is blocked, reduced sympathetic activity and enhanced parasympathetic activity are expected 8. Regarding the ACE polymorphism, animals harboring 3 copies of the ACE gene, which is accompanied by increased synthesis of Ang II, demonstrated left ventricular hypertrophy, higher pressure values, overt proteinuria, and risk of nephropathy, which is probably associated with autonomic imbalance 9-11. However, no RCTs or even experimental studies have described the relationship between autonomic regulation and ACE gene polymorphism in DM. This relationship, however, deserves attention, as renal RAS is clearly activated in DM 12.

Therefore, this study aimed to investigate hemodynamic and autonomic control as well as the renal expression level of Ang II after induction of diabetes in genetically modified mice harboring a different number of copies of the ACE gene.

Since RAS activity increases sympathetic afferent signals in response to renal ischemia, which in turn causes increased central sympathetic outflow 13, we hypothesized that in the presence of increased Ang II in the kidney, autonomic dysfunction would be proportionally augmented based on the number of ACE gene copies.

## METHODS

### Production of transgenic mice

Genetically engineered mice in which the ACE gene was either inactivated or duplicated at its endogenous locus on chromosome 11 were developed by using a gene recombination technique previously described by Krege et al. 14. Briefly, this technique can be used to insert a sequence of DNA by recombination in place of the gene to be turned off (knockout) or insert an extra copy of a gene adjacent to the existing gene (knock in).

### Genotyping

Genetically modified offspring were identified at 21 days of age by PCR amplification of DNA isolated from ear biopsies as previously described 14.

After birth, the animals are identified by a chip, an electronic device inserted subcutaneously in the back during anesthesia. Each device contains a code with letters and numbers that can be identified by telemetry through an automatic reader. The procedure is simple because it requires no sutures, and the animal fully recovers within minutes.

### Experimental groups

Experiments were performed on male mice developed by Krege et al. 14 (15-20 grams) harboring 2 and 3 copies of the ACE gene at the central animal house of the Medical School of University of São Paulo in São Paulo, Brazil. Mice were fed standard laboratory chow and water ad libitum. The animals were housed in collective polycarbonate cages in a temperature-controlled room (22-23° C) and under 54-55% humidity with a 12-h dark–light cycle (light 07:00-19:00 h). The experimental protocol was approved by the University of Sao Paulo Ethics Committee (protocol number 1226/06) and carried out according to the Declaration of Helsinki on Ethical Principles (as revised in Brazil in 2013).

The animals were randomly assigned into four groups: mice harboring 2 (2C) (n=6) and 3 (3C) (n=6) copies of the ACE gene, and STZ-induced diabetic mice with those genotypes [2CD (n=6) and 3CD (n=6)].

### Induction of diabetes

Diabetes was induced in 6-10-week-old male mice by a single intraperitoneal injection of streptozotocin (STZ, 150 mg/kg, Sigma Chemical Company, St. Louis, MO, USA) after a 12-hour fasting period. The non-diabetes group received only a vehicle (10 mM citrate buffer, pH 4.5) injection after a similar fasting period.

### Measurement of blood glucose levels

After a 24-hour fasting period, blood glucose levels were determined using the ACCU-CHECK Sensor^®^ (Roche). Blood samples were obtained by vessel puncture in the caudal vein and were used to estimate glucose levels. Animals with plasma glucose levels higher than 250 dl/ml were included in the diabetic groups. At the end of the experiments, plasma glucose was measured, and the mean of initial and final measurements was presented.

### Glucose tolerance test

A glucose tolerance test (GTT) was performed at the end of the 30-day protocol in all study groups. The animals fasted for 6 hours and were given a single (i.p.) injection of glucose (1.5 g/kg). Blood samples were collected from a small cut made in the tail of each animal at 0 (zero), 5, 15, 30, 60 and 90 min after glucose load, and the area under the curve (AUC) was calculated. Blood glucose concentrations were determined using an Accu-Check Advantage Blood Glucose Monitor (Roche Diagnostic Corporation, Indianapolis, IN).

### Cardiovascular measurements

One day after the GTT, at 31 days after diabetes induction, the animals were anesthetized (ketamine–xylazine 80:40 mg/kg i.p.), and polyethylene-tipped Tygon cannulas (4 cm of PE-08 connected to 2 cm of PE-50, Clay Adams) filled with heparinized saline were inserted into the carotid artery and jugular vein for direct measurements of arterial pressure (AP) and drug administration, respectively. The free ends of the cannulas were tunneled subcutaneously and exteriorized at the top of the skull. After surgery, the animals received an intramuscular injection of penicillin G (Benzetacil^®^, Fontoura-Wyeth, 60,000 U). Two days following catheter placement, hemodynamic measurements were taken from animals at baseline conditions, namely, conscious, freely moving, and in a quiet environment with controlled temperature but deprived of food and water. The arterial cannula was connected to a transducer (Blood Pressure XDCR, Kent Scientific), and AP signals were recorded for a 20-min period using a microcomputer equipped with an analog-to-digital converter board (WINDAQ, 4 kHz, Dataq Instruments). The recorded data were analyzed on a beat-to-beat basis to quantify changes in AP and heart rate (HR).

### HR and blood pressure variability

Time-domain analysis was carried out by calculating the mean pulse interval (PI) variance using its respective time series. For the frequency domain analysis, the whole 30-min time series of PI and systolic arterial pressure (SAP), which totaled 256 points with a 50% overlap, was used. Following linear trend removal, power spectral density was obtained by fast Fourier transformation. Spectral power for low (LF 0.10-1.0 Hz) and high (HF 1-5 Hz) frequency bands was chosen using the methods described by Thireau 15 to calculate spectral components by the mean of the power spectrum density integration within each frequency bandwidth using a customized routine (MATLAB 6.0, Mathworks).

### Assessment of renal angiotensin II

At the end of the study period, the animals were anesthetized with an overdose of pentobarbital 120 mg/kg (Critália, Sao Paulo, Brazil), and nephrectomy was performed. The right kidney was perfused in situ and prepared for immunohistochemical evaluation. Perfusion of the kidney was first carried out using a saline solution to remove red blood cells from the vascular lumen followed by a 10% formalin solution for 3 minutes to preserve the structures. The kidney was sectioned in the coronal orientation at approximately 4 mm thickness. We measured Ang II expression levels in the renal cortex. For this quantification, we counted the number of positively stained cells in 25 consecutive microscopic fields, and the results were expressed as the mean number of stained cells per square millimeter. The fragments were kept for 2 hours in a formaldehyde solution plus 10% phosphate buffer and embedded in paraffin blocks.

For the immunohistochemical analysis, tissues were deparaffinized and then incubated with the specific primary antibody against angiotensin II (Anti-Ang II-rabbit polyclonal Peninsula, Belmont, CA, USA) and with biotinylated immunoglobulin goat anti-rabbit antibody (Vector, Burlingame, USA) diluted 1:1,000 for 45 minutes. Additionally, a complex of biotin-streptavidin/alkaline phosphatase (Vector, Burlingame, USA) was added for 30 minutes.

The antigen was stained by fast red dye, and the specificity of the secondary antibody was established in positive and negative controls. Images were obtained with a computer-assisted morphometric system, and the number of positive cells was expressed as Ang II/mm^2^.

### Statiscal Analysis

Statistical analyses were performed using SPSS software (Version 17.0 for Windows; SPSS Inc., Chicago, USA). Data are reported as the mean±SEM. After confirming that all continuous variables were normally distributed using the Kolmogorov–Smirnov test, significant differences between the groups were obtained by two-way ANOVA followed by the Tukey post-test. All tests were two sided, and statistical significance was established at *p*<0.05.

## RESULTS

### Glucose levels and hemodynamic parameters

Diabetic animals showed higher plasma glucose levels as well as lower tolerance to glucose than control animals, regardless of the number of ACE copies (i.e., 2C and 3C) (Table 1). Regarding hemodynamic parameters, there were no differences in blood pressure values (i.e., systolic, diastolic and mean) between the groups; however, a lower HR was detected in diabetic animals than in control animals (Table 1).

### Autonomic modulation

HR variability, expressed by pulse interval variability (PIV) in time and frequency domains, is shown in Table 2. No difference in PI variance was observed between the groups, thus indicating that diabetes and the number of ACE gene copies did not change the PIV. However, the 3CD group showed increased low-frequency bands (%LF) and reduced high-frequency bands (%HF) of the PIV, which resulted in increased sympathovagal balance (LF/HF) in the 3CD group when compared to all experimental groups.

### Angiotensin in renal cortex

**Figure 1:**
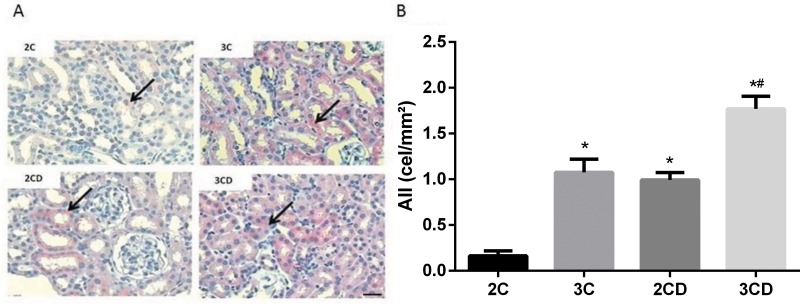
Quantification of angiotensin II expression levels in the renal cortex of 2C, 3C, 2CD and 3CD mice by immunohistochemical staining: (A) Illustrative representation of angiotensin II expression in the experimental groups; (B) bar graph of angiotensin II expression levels as determined by immunohistochemical staining. The arrows indicate positive angiotensin II staining. Bar: 400X. *p<0.05 *vs*. the 2C control group, and #*p*<0.05 *vs.* all groups.

Figure 1 shows the expression of Ang II in the renal cortex. Data demonstrated an increase in positive Ang II staining in the renal tubules of the 3C, 2CD and 3CD groups. Moreover, Ang II was overexpressed in the 3CD group compared to the other groups.

### Correlation

**Figure 2:**
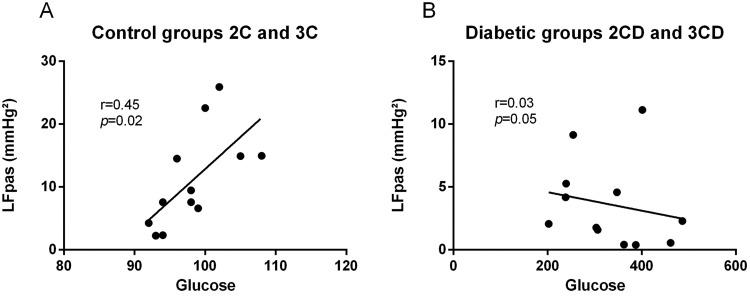
Linear regression analysis of LF SAP and glucose: (A) in control groups (2C and 3C) and (B) in diabetic groups (2CD and 3CD).

Correlations were identified between the increase in LFpas and increase in glucose levels in the control group regardless of the number of ACE copies (i.e., 2C and 3C) (r=0.72, *p*<0.02) (Figure 2A). On the other hand, no correlations were found between diabetic groups and LFpas (r=0.03, *p*<0.05) (Figure 2B).

## DISCUSSION

The present study was the first to demonstrate that STZ-induced diabetic mice with 3 copies of the ACE gene presented increased sympathetic modulation and reduced vagal modulation of the heart. It is important to mention that to the best of our knowledge, this was the first study to show that autonomic modulation may be affected by the number of ACE gene copies in DM.

Conflicting results were found in the literature regarding the variation of RAS in DM 16. These discrepancies may be attributed to the different animal models used, the diseases state and progression, fluctuation levels of the genetically determined components of RAS and glucose levels reached during illness 17. ACE gene polymorphisms might also account for these discrepancies since ACE gene variance is associated with increased risk of glucose intolerance in the healthy subjects 18.

Regarding the clinical parameters associated with the diabetic condition, as expected, diabetic animals showed increased baseline plasma glucose as well as an increased AUC in the GTT. Ang II seems to interact with insulin metabolism, as the 3C group showed higher insulin resistance than the 2C group. Finally, the ACE genotype did not affect hyperglycemia.

These findings may be explained by the fact that Ang II promotes insulin resistance by interfering in the insulin-stimulated increase in insulin receptor substrate 1-(IRS-1)-associated PI3K activity 24. In addition, reduced insulin sensitivity was found in both animals with 3 copies of the ACE gene and healthy humans with the D allele 18,25. The ANS and the kidneys crosstalk through renal nerves. Indeed, Ang II facilitates neurotransmission by enhancing sympathetic discharge and increasing the arterial pulse, which increases ganglionic transmission 26. This function of Ang II is well illustrated by renal denervation, when the increase in blood pressure is prevented in models of induced hypertension 27.

Efferent renal sympathetic nerves may modify renal vascular resistance. In addition, afferent renal nerves are responsible for carrying information from the renal baroreceptor to the central nervous system 28, thus altering afferent sympathetic nervous discharge. Therefore, afferent sympathetic nervous discharge may be modulated by an increase in Ang II expression, as demonstrated in the present study. Moreover, afferent sympathetic nervous discharge may impact the central sympathetic tonus and, consequently, increase sympathetic modulation of the heart in diabetic mice. Indeed, some studies have shown 26 that increased Ang II expression in the kidney may damage the central sympathetic tonus in DM.

On the other hand, Ang II in the kidney may facilitate the release of noradrenaline from the renal sympathetic nerve terminal, thus changing the hemodynamic parameters and contributing to systemic changes in blood pressure control 26.

In the present study, animals were genetically modified to model the changes and fluctuations in RAS commonly observed in humans. In fact, this animal model has been accepted as a useful tool with which to investigate the effect of vascular and renal ACE on hemodynamic parameters 14. Regarding hemodynamic parameters, diabetic animals showed reduced AP levels, and these data are in accordance with other evidence in the literature 19. However, as previously described by studies conducted by our group 11 and other groups 14, no significant difference in AP was observed among mice with 2 (i.e., ACE2) and 3 (i.e., ACE3) copies of the ACE gene at the end of the study regardless of the diabetic condition. A possible explanation of this phenomenon is the inherently elevated homeostatic capacity presented by these animals; however, it is important to mention that in some stressful situations, differences in blood pressure levels may be observed in this experimental model 20.

Hemodynamic analyses also indicated rest bradycardia in diabetic groups compared with non-diabetic groups. Evidence in the literature has demonstrated rest bradycardia and impaired cardiovascular reflexes in experimental diabetes animals 21. This finding may be explained by the fact that the high number of ACE gene copies are associated with increased Ang II levels, which may trigger additional changes in intrinsic HR. In this sense, although decreased HR in diabetic animals has been attributed to changes in the sinoatrial node, functional alterations in the cholinergic mechanism cannot be ruled out 22.

Autonomic modulation analysis demonstrated dissimilar results between diabetic and non-diabetic animals. In non-diabetic animals, the analysis of PIV in the frequency domain shows that the normalized LF component of PIV, which represents the sympathetic modulation to the heart 23, was not different between the animals with 2 and 3 copies of the ACE gene, even if different ACE activity was observed in these animals. However, in 3CD diabetic animals, it was possible to observe an increased LF band of HR when compared with all other studied groups. These results indicate that the diabetic state associated with high levels of Ang II elicited by an elevated number of copies of the ACE gene may increase cardiac sympathetic modulation.

Last, we explored a possible association between the peripheral sympathetic component and glucose levels and found a positive correlation in the control groups (r=0.72, *p*<0.02) but not in the diabetic groups (r=0.03, *p*<0.05). Although increased LFpas component was found to be normal in diabetic groups, in the present study, the diabetic groups showed reduced LFpas throughout the diabetes time-course, thus compromising the peripheral nerve and reducing its response, as previously reported in the literature 29.

The amount of Ang II in the renal cortex of diabetic groups, as evaluated by immunohistochemistry, may be associated with the relationship between hyperglycemia and increased RAS activity 30,31. Furthermore, our data show that the increase in Ang II expression was proportional to the number of copies of the ACE gene, since the increase in Ang II expression was higher in the 3CD group than in the 2CD group. These findings suggest that animals with more ACE gene copies exhibit increased activation of renal RAS and that this increase is potentiated by diabetes.

Indeed, the increase in the intrarenal formation of Ang II may change the glomerular hemodynamics 32, as suggested by Huang et al., 10 whom found several physiopathological alterations in the renal parameters of diabetic animals with 3 copies of the ACE gene, such as microalbuminuria.

Our data indicate that a small increase in ACE activity in diabetic animals due to increased ACE gene copies leads to greater impairment of autonomic function, as demonstrated by increased sympathetic modulation and reduced cardiac vagal modulation along with increased renal expression of Ang II in these groups. Taken together, these results reinforce the contribution of RAS activation to the development of cardiovascular and renal changes observed in diabetes.

## AUTHOR CONTRIBUTIONS

Moraes OA, Flues K, De Angelis K and Irigoyen MC designed the study. Moraes OA and Flues K were responsible for project coordination. Moraes OA, Flues K, Dartora DR and Scapini KB were responsible for the data collection. Evangelista FS performed the immunohistochemical evaluation. Mostarda C, Rodrigues B and Fiorino P were responsible for cardiac autonomic modulation and statistical analyses. Moraes OA, Flues K, Dartora DR and Scapini KB drafted the manuscript. Mostarda C, Evangelista FS, Rodrigues B, Fiorino P, De Angelis K and Irigoyen MC revised the manuscript. All authors have approved the submitted version of the manuscript.

## Figures and Tables

**Table 1:** Metabolic data and hemodynamic evaluation of mice harboring 2 (2C) and 3 (3C) copies of the ACE gene and STZ-induced diabetic mice with the same genotype (2CD and 3CD).

Parameters	2C	3C	2CD	3CD
**Glycemia (mg/dL)**	99±7	97±2	347±34*^†^	317±62*^†^
**GTT (mg/dL/min)**	219±15	278±45*	491±19*^†^	452±42*^†^
**SAP (mmHg)**	122±3.2	125±4	122±4	123±3
**DAP (mmHg)**	86±4.1	90±5	93±5	94±4
**MAP (mmHg)**	106±3.1	107±2	102±5	104±5
**HR (bpm)**	611±14	596±25	467±23*^†^	513±12*^†^

Values are expressed as the mean±SEM. DAP: diastolic arterial pressure; GTT: glucose tolerance test; HR: heart rate; MAP: mean arterial pressure; and SAP: systolic arterial pressure. *p<0.05 *vs*. 2C; and ^†^*p*<0.05 *vs*. 3C.

**Table 2:** Autonomic assessment in mice harboring 2 (2C) and 3 (3C) copies of the ACE gene and STZ-induced diabetic mice with the same genotype (2CD and 3CD).

Variables	2C	3C	2CD	3CD
**PIV (ms)**	38±8	30±5	20±6	28±5
**LF (ms^2^)**	4.6±1	4.8±1	1.8±0.5	16.9±5*^#+^
**HF (ms^2^)**	9.9±1.5	9.2±1.1	3.6±6	12.0±6
**%LF**	31±4	33±7	37±6	60±7*^#^
**%HF**	69±4	67±7	63±6	40±7*^#^
**LF/HF**	0.48±0.1	0.56±0.2	0.68±0.2	2.20±0.7*

Values are expressed as the mean±SEM. PIV: pulse interval variability; LF: absolute values of the low-frequency band of the pulse interval; HF: absolute values of the high-frequency band of the pulse interval; %LF: normalized low-frequency band; %HF: normalized high-frequency band; and LF/HF ratio: ratio of absolute values of low-frequency and high-frequency bands. *p<0.05 *vs*. all; #*p*<0.05 *vs*. 3C; and +*p*<0.05 *vs*. 2CD.
